# A Ras destabilizer KYA1797K overcomes the resistance of EGFR tyrosine kinase inhibitor in *KRAS*-mutated non-small cell lung cancer

**DOI:** 10.1038/s41598-018-37059-8

**Published:** 2019-01-24

**Authors:** Jieun Park, Yong-Hee Cho, Wook-Jin Shin, Sang-Kyu Lee, JaeHeon Lee, Taehyung Kim, Pu-Hyeon Cha, Jee Sun Yang, Jaebeom Cho, Do Sik Min, Gyoonhee Han, Ho-Young Lee, Kang-Yell Choi

**Affiliations:** 1 Translational Research Center for Protein Function Control, Yonsei University, Seoul, Korea; 2 Department of Biotechnology, College of Life Science and Biotechnology, Yonsei University, Seoul, Korea; 3 Department of Molecular Biology, College of Natural Science, Pusan National University, Pusan, Korea; 4 College of Pharmacy and Research Institute of Pharmaceutical Sciences, Seoul National University, Seoul, Korea

## Abstract

The epidermal growth factor receptor (EGFR) inhibitors such as erlotinib and gefitinib are widely used for treatment of non-small cell lung cancer (NSCLC), but they have shown limited efficacy in an unselected population of patients. The *KRAS* mutations, which are identified in approximately 20% of NSCLC patients, have shown to be associated with the resistance to the EGFR tyrosine kinase inhibitors (TKIs). Currently, there is no clinically available targeted therapy which can effectively inhibit NSCLC tumors harboring *KRAS* mutations. This study aims to show the effectiveness of KYA1797K, a small molecule which revealed anti-cancer effect in colorectal cancer by destabilizing Ras via inhibiting the Wnt/β-catenin pathway, for the treatment of *KRAS*-mutated NSCLC. While erlotinib fail to have anti-transforming effect in NSCLC cell lines harboring *KRAS* mutations, KYA1797K effectively inhibited the Ras-ERK pathway in *KRAS*-mutant NSCLC cell lines. As a result, KYA1797K treatment suppressed the growth and transformation of *KRAS* mutant NSCLC cells and also induced apoptosis. Furthermore, KYA1797K effectively inhibited *Kras*-driven tumorigenesis in the *Kras*^*LA2*^ mouse model by suppressing the Ras-ERK pathway. The destabilization of Ras via inhibition of the Wnt/β-catenin pathway is a potential therapeutic strategy for *KRAS*-mutated NSCLC that is resistant to EGFR TKI.

## Introduction

Lung cancer is the leading cause of cancer death constituting the highest percentage of cancer mortality worldwide^1,2^ and non-small cell lung cancer (NSCLC) is the most common histological subtype of lung cancer. Since NSCLC is composed of many genomic subsets, each with its own driver mutation, research efforts were made to develop therapeutic drugs that selectively target a specific driver mutation such as *KRAS*, epidermal growth factor receptor (*EGFR*), *BRAF*, and anaplastic lymphoma kinase (*ALK*). Erlotinib (Tarceva®) and gefitinib (Iressa®), EGFR tyrosine kinase inhibitors (TKIs), have been developed and widely used for treatment of NSCLC patients, yet their effectiveness was limited to the selected population of patients with EGFR abnormalities^3^. Crizotinib (Xalkori®), which specifically targets and inhibits kinase activity of EML4-ALK, has been developed as a targeted therapy for NSCLC patients with *EML4-ALK* fusion oncogenes, which account for 3 to 7% of NSCLC mutations^4,5^. These molecular targeted therapies, each of which specifically targets one driver mutation, brought clinically meaningful outcomes in treating NSCLC^6,7^. However, the development of specific and potent inhibitor of *KRAS* has not been accomplished although *KRAS* mutation accounts for more than 20% of all NSCLC mutations^8^.

Approximately 80% of lung cancers are NSCLC^9^, and Ras signaling pathway is activated in nearly half of NSCLC patients due either to amplification of *EGFR* or to activating mutations in *EGFR* or *KRAS*^3^. For patients with amplified *EGFR* or activating mutations in *EGFR*, the treatment of EGFR TKIs such as gefitinib and erlotinib has brought remarkable benefits, resulting in patients’ prolonged progress-free survival and overall survival^7^. However, many patients treated with the EGFR TKIs inevitably developed therapeutic resistance^10^ and more importantly, patients with activating mutation in *KRAS* exhibited primary resistance to the treatment of EGFR TKIs^11^. In addition, a large number of patients who initially responded to EGFR TKI eventually acquired resistance due to secondary mutation in the *EGFR* gene (T790M mutation)^12^. Thus, despite its striking efficacy, EGFR TKIs are effective only in a subset of NSCLC patients with EGFR abnormalities and the duration of its action is short.

EGFR TKIs inhibit the Ras-Raf-MEK-ERK signaling cascades by blocking the catalytic activity of EGFR, yet they cannot block the signaling cascades in the presence of *KRAS* mutation since Ras is the downstream effector of EGFR. Although Ras remains to be one of the most attractive targets for various human cancers including NSCLC, there is no clinically available anti-cancer drug targeting Ras, which is often considered as an undruggable target^13^. As an effort to control Ras protein, we recently developed and characterized small molecules showing anti-cancer effect in colorectal cancer (CRC) through degradation of Ras via targeting the Wnt/β-catenin pathway^14,15^. KYA1797K, one of the compounds that inhibited transformation of CRC cells harboring mutant *KRAS*, directly binds to the N-terminal regulators of the G-protein signaling (RGS) domain of Axin and enhances the formation of the β-catenin destruction complex resulting in GSK3β activation^14^. Activated GSK3β phosphorylates Ras and β-catenin and leads to the subsequent recruitment of β-TrCP E3 ligase. As a result, both Ras and β-catenin undergo polyubiquitinylation-dependent proteasomal degradation^14^. We hypothesized that KYA1797K, which destabilizes Ras via suppression of the Wnt/β-catenin pathway, may suppress growth of NSCLC tumors with aberrant activation of the EGFR-Ras-ERK pathway, including those resistant to EGFR TKIs due to *KRAS* mutations. The rationale for this novel approach to control cancer via small molecule-mediated Ras degradation was further strengthened by our observation that both β-catenin and RAS are overexpressed in NSCLC patient tissues and the results of recent studies that suggest approaches degrading target proteins as a promising anti-cancer therapeutic strategy in cancer^16^. We also predicted that the use of KYA1797K for the treatment of NSCLC will provide an additional advantage by inhibiting the Wnt/β-catenin pathway since activation of the Wnt/β-catenin pathway promotes hyper-proliferation of lung cancer cells and inhibition of the Wnt/β-catenin pathway synergizes the effect of EGFR inhibition^17–19^. In addition, a recent study identified that the Wnt/β-catenin pathway is one of the underlying pathways causing NSCLC relapse after treatment of EGFR-driven NSCLC with EGFR inhibitors, such as gefitinib and erlotinib, since the Wnt/β-catenin pathway works as a mechanism of protection from EGFR inhibition^20^. We also revealed that aberrant Wnt/β-catenin signaling activates cancer stem cells when oncogenic *KRAS* mutations is present in colorectal cancer^21^. Therefore, drugs that suppress EGFR-KRAS pathway via inhibition of the Wnt/β-catenin pathway, such as KYA1797K, are expected to be an effective therapy for the treatment of EGFR-driven NSCLC.

To validate our hypothesis, we used five NSCLC cell lines harboring either wild-type or mutant *KRAS* and addressed the effect of *KRAS* mutations on the responsiveness of these cell lines to erlotinib. Erlotinib effectively suppressed the growth and colony formation of *KRAS* wild-type NSCLC cell lines but not of *KRAS* mutant cell lines, confirming the resistance of EGFR-targeted therapy in *KRAS* mutated NSCLC. We then investigated the effect of KYA1797K on these NSCLC cell lines to find out if KYA1797K could overcome the resistance of *KRAS* mutated NSCLC to erlotinib and observed that KYA1797K successfully overcomes the resistance of erlotinib in *KRAS* mutant cell lines. In both *KRAS* wild-type and mutant cell lines, KYA1797K effectively inhibited the growth and colony formation. We demonstrated that KYA1797K also has *in vivo* anti-cancer effect using *Kras*^*LA2*^ mouse model^22^, which has shown to be highly disposed to early onset lung cancer. While erlotinib failed to inhibit *Kras-*driven tumorigenesis in *Kras*^*LA2*^ mice, KYA1797K treatment successfully reduced both the number and size of tumors formed by *Kras* mutations through effective suppression of the Ras-ERK pathway. Overall, this study suggests the use of a small molecule KYA1797K, which destabilizes both Ras and β-catenin, as a novel targeted therapeutic strategy that overcomes the resistance of EGFR TKIs in *KRAS* mutated NSCLC.

## Results

### Both β-catenin and pan-Ras are overexpressed in NSCLC and *KRAS* mutant NSCLC cell lines exhibit resistance to erlotinib

**Figure 1:**
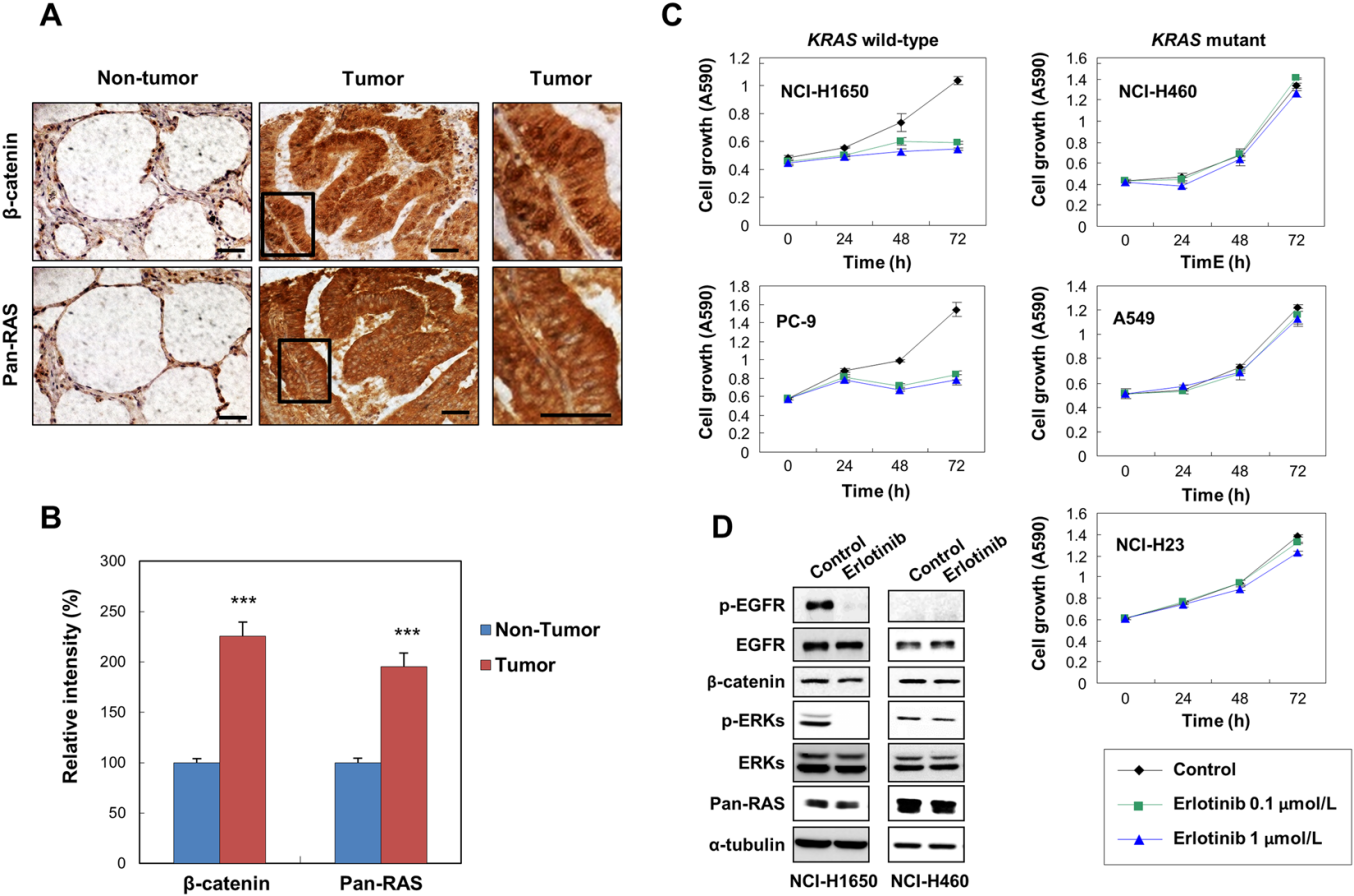
Expression of β-catenin and pan-Ras in NSCLC and effects of erlotinib on proliferation, the EGFR-Ras-ERK and the Wnt/β-catenin pathways in NSCLC. (**A**) DAB immunohistochemical analyses of β-catenin and pan-Ras expression of patient tissue samples on a tissue microarray, which contains 75 cores of histologically confirmed NSCLC samples and 75 cores of non-tumor lung samples (75 matched pairs). Scale bars, 20 μm. (**B**) Expression of β-catenin and pan-Ras were quantified using NIS-Elements AR3.2 program (Nikon). Relative intensity is normalized to non-tumor and data represent mean ± SEM. ^***^P < 0.001; *n* = 75. (**C**) MTT assays were carried out at 0, 24, 48, and 72 hours after the treatment of erlotinib in the five NSCLC cell lines. The error bars represent the SD; *n* = 3. (**D**) Cells were harvested 24 hours after treatment of 1 μmol/L erlotinib in NCI-H1650 and NCI-H460 cells and whole cell lysates (WCLs) were subjected to the immunoblot analyses using indicated antibodies.

In order to investigate if destabilization of both Ras and β-catenin would be a therapeutic strategy for the treatment of NSCLC, we checked whether Ras and β-catenin are overexpressed in tumor regions compared to non-tumor regions. The levels of both Ras and β-catenin were increased in NSCLC tumor regions compared to adjacent normal regions as shown by DAB staining of the histologically confirmed 75 NSCLC tissue specimens on the tissue microarray (Fig. 1A,B). To study if *KRAS* mutation can affect the responsiveness of each NSCLC cell line to erlotinib, an EGFR TKI, we divided five NSCLC cell lines of different genetic backgrounds (Supplementary Table 1) into two groups according to their *KRAS* mutation status. Treatment of erlotinib for 72 hours suppressed proliferation of *KRAS* wild-type cell lines, NCI-H1650 and PC-9^23,24^, by 47% and 44%, respectively (Fig. 1C). On the other hand, NCI-H460, A549 and NCI-H23 cells, all of which harbor *KRAS* mutation, clearly exhibited resistance to erlotinib as their proliferation was not inhibited by erlotinib at the same concentration (Fig. 1C).

We performed immunoblot analyses to confirm that erlotinib treatment inhibits the Ras-ERK pathway in *KRAS* wild-type cells but not in *KRAS* mutant cells using NCI-H1650 and NCI-H460 cells. Erlotinib treatment strongly suppressed ERK activities in *KRAS* wild-type NCI-H1650 cells, while it only mildly inhibited ERK activities in *KRAS* mutant NCI-H460 cells (Fig. 1D). The levels of β-catenin as well as pan-Ras were not changed by erlotinib treatment regardless of *KRAS* mutational status. However, treatment of erlotinib resulted in the reduction of phospho-EGFR (p-EGFR), representing activation status of EGFR^25^, in NCI-H1650 cells (Fig. 1D).

### KYA1797K inhibits the Ras-ERK pathway, proliferation, and colony formation of erlotinib-resistant *KRAS* mutant NSCLC cell lines by destabilizing Ras

**Figure 2:**
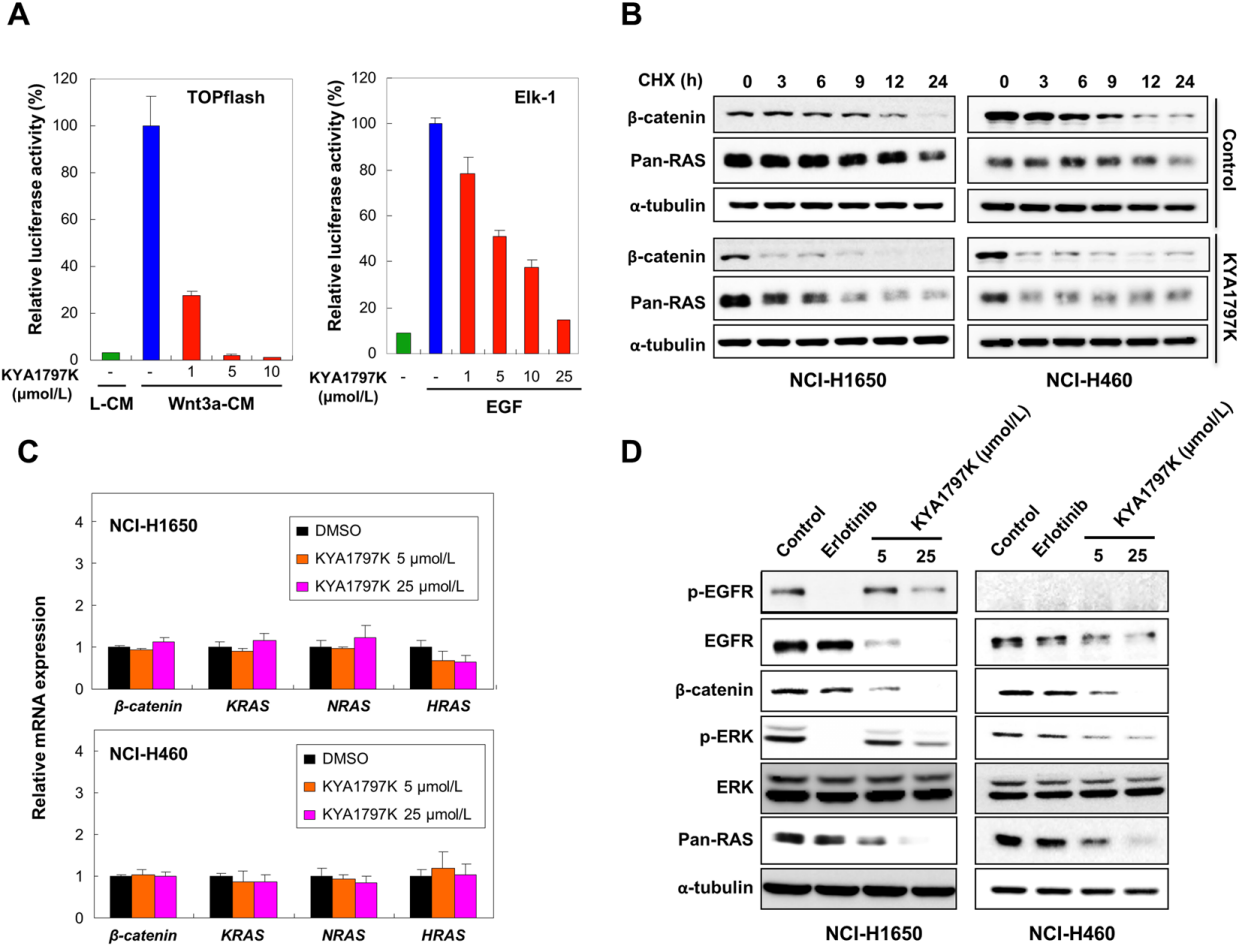
Effects of KYA1797K on the Wnt/β-catenin and the EGFR-Ras-ERK pathways in *KRAS* WT and MT NSCLC cell lines. (**A**) TOPflash reporter and Elk-1 reporter activities were measured after treating KYA1797K for 24 hours. Media for HEK-TOP cells were changed to either L-cell conditioned media (L-CM) or Wnt3a-CM with 1, 5, or 25 μmol/L of KYA1797K treatment (TOPflash). HEK-293 cells were treated with EGF along with indicated dose of KYA1797K for 24 hours (Elk-1). Error bars indicate the SD; *n* = 3. (**B**) NCI-H1650 and NCI-H460 cells were treated with 25 μmol/L KYA1797K along with cycloheximide (50 μg/mL) and were harvested at different time points after the treatment. (**C**,**D**) NCI-H1650 and NCI-H460 cells were harvested 24 hours after the treatment of 1 μmol/L erlotinib, 5 or 25 μmol/L KYA1797K. Total RNAs were isolated and subjected to the real time reverse transcription-PCR (**C**) or whole cell extracts were prepared for immunoblot analyses (**D**). Relative mRNA expression is represented by normalization to DMSO-treated control and is presented as mean ± SD; *n* = 3 (**C**).

KYA1797K inhibited both the Wnt/β-catenin and the Ras-ERK pathways in a dose-dependent manner as shown by inhibitions of the TOPflash and Elk-1 reporter activities (Fig. 2A). To investigate if KYA1797K suppresses the Wnt/β-catenin and the Ras-ERK pathways via destabilizing β-catenin and Ras proteins^14^, we treated KYA1797K along with cycloheximide, a protein synthesis inhibitor, to *KRAS* wild-type NCI-H1650 and *KRAS* mutant NCI-H460 cell lines. KYA1797K treatment accelerated reduction of β-catenin and pan-Ras levels in both NCI-H1650 and NCI-H460 cells (Fig. 2B). The reduction of pan-Ras by KYA1797K can be attributed to destabilization of Ras proteins^14^, not to regulation of Ras at the transcriptional level (Fig. 2C). KYA1797K effectively lowered ERK activity as well as protein levels of β-catenin and pan-Ras in both NCI-H1650 and NCI-H460 cells (Fig. 2D). On the contrary, erlotinib inhibited ERK activity only in *KRAS* wild-type NCI-H1650 cells and did not affect protein levels of β-catenin or pan-Ras in either cell lines (Fig. 2D). Erlotinib reduced p-EGFR level without significantly changing EGFR levels in NCI-H1650 cells, as expected (Fig. 2D). Interestingly, levels of both EGFR and p-EGFR were significantly reduced by KYA1797K (Fig. 2D), and this may be attributed to the fact that EGFR is a transcriptional target of Wnt/β-catenin pathway^26^. p-EGFR was not detected in NCI-H460, possibly due to the low basal activity of EGFR in this cell line^27^.

**Figure 3:**
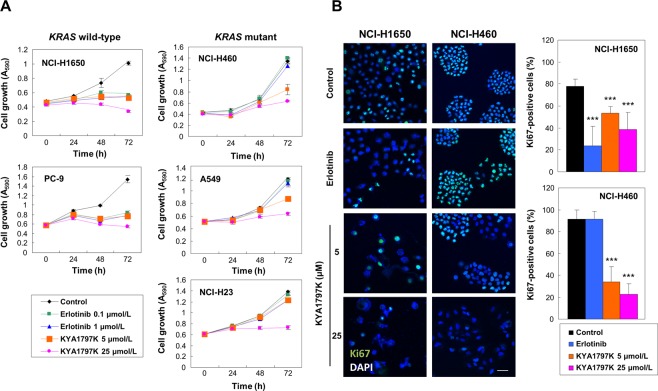
Effects of KYA1797K on cell growth and proliferation of *KRAS* WT and MT NSCLC cell lines. Cells were treated with erlotinib or KYA1797K. (**A**) MTT assays were done at 0, 24, 48, and 72 hours after the treatment of erlotinib or KYA1797K. Error bars represent the SD; *n* = 3. (**B**) NCI-H1650 and NCI-H460 cells treated with 1 μmol/L erlotinib, 5 or 25 μmol/L KYA1797K were stained for Ki67 and nuclei were counter stained with DAPI. The percentage of Ki67-positive cells was calculated from the total number of DAPI-stained cells, and error bars indicate the SD. ^*^P < 0.05, ^**^P < 0.01, ^***^P < 0.001; *n* = 3. Scale bar, 40 μm.

**Figure 4:**
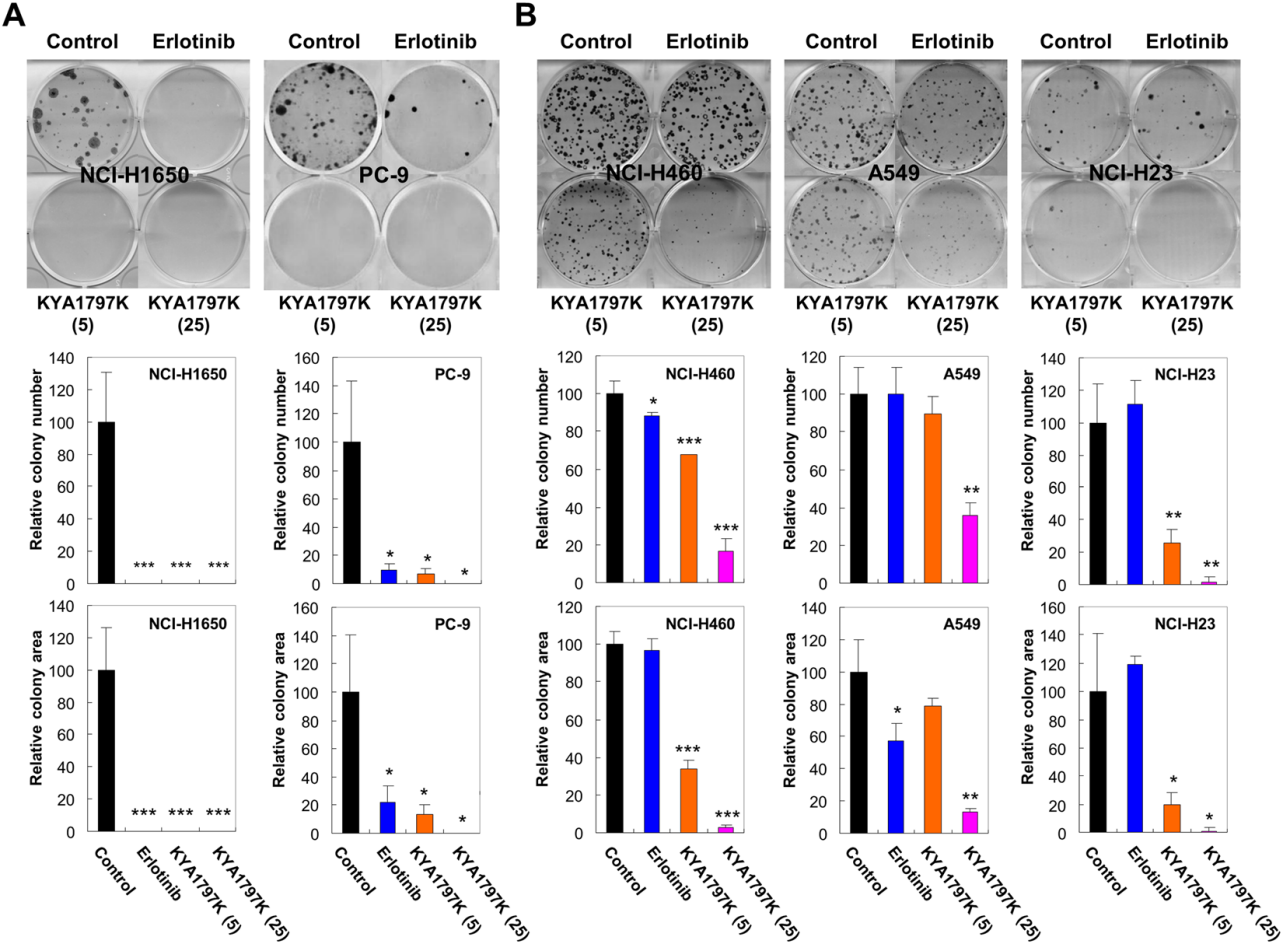
Effects of KYA1797K on colony formation capacity of *KRAS* WT and MT NSCLC cell lines. (**A**,**B**) *KRAS* WT (**A**) and *KRAS* MT (**B**) NSCLC cell lines were treated every 2 or 3 days with 1 μmol/L erlotinib, 5 or 25 μmol/L KYA1797K for 10–21 days. The foci were photographed using a bright-field optical microscope. Quantitative data for the relative percentages of colony numbers and areas are determined with ImageJ software and represented at the bottom, with error bars indicating the SD. ^*^P < 0.05, ^**^P < 0.01, ^***^P < 0.001; *n* = 3.

Effects of KYA1797K on cellular proliferation were measured using MTT assay (Fig. 3A) and Ki67 staining (Fig. 3B). Treatment with erlotinib reduced proliferation of *KRAS* wild-type cell lines, but not of *KRAS* mutant cell lines (Fig. 3A). However, KYA1797K suppressed cell growth of both *KRAS* wild-type and mutant cell lines (Fig. 3A), without notable differences in IC_50_ values across cell lines (Supplementary Fig. S1). Ki67 staining also showed that erlotinib treatment reduces proliferation of only *KRAS* wild-type cell line while KYA1797K treatment reduces proliferation both *KRAS* wild-type and mutant cell lines (Fig. 3B). Erlotinib treatment reduced the percentage of Ki67-positive cells by 54% in *KRAS* wild-type NCI-H1650 cells, but had no effect on proliferation in *KRAS* mutant NCI-H460 cells (Fig. 3B). On the other hand, KYA1797K treatment resulted in the reduction of Ki67-positive cells in both *KRAS* wild-type NCI-H1650 cells and *KRAS* mutant NCI-H460 cells by 39% and 70%, respectively (Fig. 3B). Consistent to the results of proliferation assays, erlotinib inhibited colony forming capacity of only *KRAS* wild-type cell lines, while KYA1797K reduced that of both *KRAS* wild-type and mutant cell lines (Fig. 4A,B). Since KYA1797K had sufficient anti-proliferation and anti-colony formation activities by itself, it was difficult to observe combinatory effect of erlotinib and KYA1797K in the NSCLC cell lines (Supplementary Figs S2 and S3).

We also compared the efficacy of KYA1797K with cisplatin and pemetrexed, which are the drugs used as a standard regimen for first-line treatment of advanced NSCLC^28^. KYA1797K showed comparable efficacy to cisplatin or pemetrexed in suppressing cell proliferation and colony formation in NCI-H460 cell line and slightly lower efficacy in A549 cell line. When KYA1797K was combined with cisplatin or pemetrexed, it had synergistic effect in suppressing cell proliferation and colony formation (Supplementary Fig. S4), suggesting that KYA1797K can be an effective targeted therapy drug candidate to be combined with standard regimen chemotherapy.

**Figure 5:**
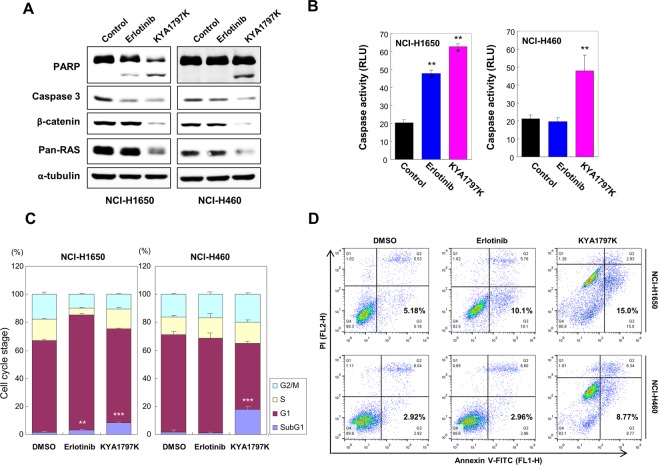
Effects of erlotinib or KYA1797K on apoptosis in *KRAS* WT NCI-H1650 and *KRAS* MT NCI-H460 cells. (**A**–**D**) Cells treated with 1 μmol/L erlotinib or 25 μmol/L KYA1797K were harvested after 36 hours (**A**,**B**) and 48 hours (**C**,**D**). (**A**,**B**) The effects of erlotinib or KYA1797K on apoptosis in these cell lines were measured by immunoblot analyses (**A**) and by caspase-glo 3/7 assay (**B**). Data represent mean ± SD; ^**^P < 0.01, ^***^P < 0.001; *n* = 3. (**C**,**D**) The effects of erlotinib and KYA1797K on cell cycle (**C**) and on apoptosis (**D**) were analyzed by PI staining and Annexin V/PI staining, respectively. Error bars represent the SD; ^**^P < 0.01, ^***^P < 0.001; *n* = 3.

Since the Ras-ERK signaling cascade is also known to play a role in inhibiting apoptosis^29^, we examined the effects of KYA1797K treatment on apoptosis through immunoblot analyses and caspase 3/7 activity assay. Treatment with erlotinib induced cleavage of PARP and increase in caspase 3/7 activity in *KRAS* wild-type NCI-H1650 cells, but not in *KRAS* mutant NCI-H460 cells (Fig. 5A,B). However, treatment of KYA1797K resulted in increase of cleaved PARP and caspase 3/7 activity in both NCI-H1650 cells and NCI-H460 cells (Fig. 5A,B), indicating induction of apoptosis upon KYA1797K treatment. The effect of KYA1797K on apoptosis was confirmed by cell cycle analysis and Annexin V/PI staining (Fig. 5C,D). Erlotinib treatment resulted in slight increase of sub-G1 phase population (Fig. 5C) and percentage of apoptotic cells (Fig. 5D) only in *KRAS* wild-type NCI-H1650 cells. On the other hand, sub-G1 phase population and percentage of apoptotic cells were increased by KYA1797K treatment in both *KRAS* wild-type NCI-H1650 and *KRAS* mutant NCI-H460 cells (Fig. 5C,D).

### KYA1797K inhibits *Kras* driven tumorigenesis in erlotinib-resistant *Kras*^*LA2*^ mice

**Figure 6:**
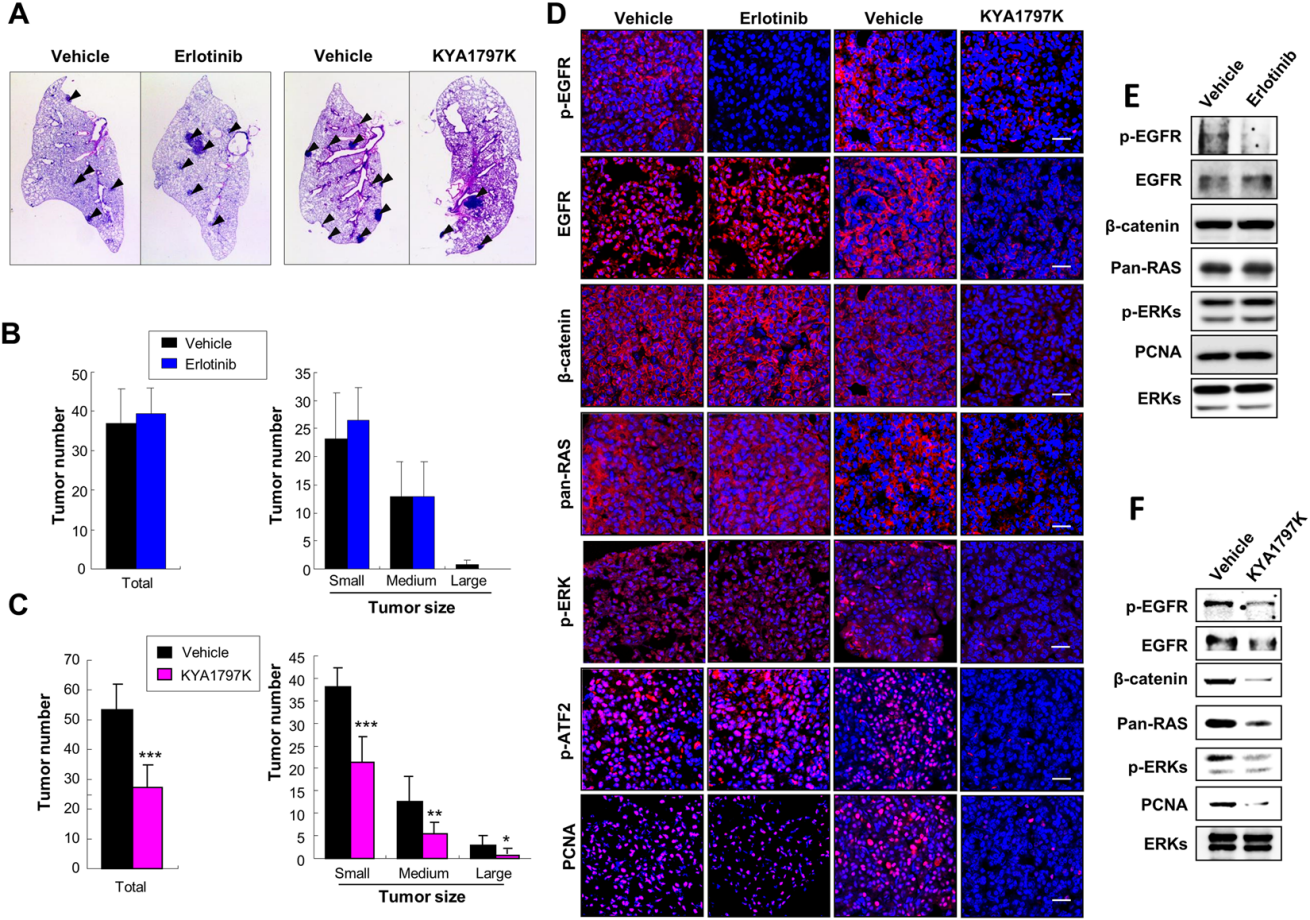
Effects of KYA1797K on lung tumorigenesis and on the Wnt/β-catenin and the Ras-ERK pathways in erlotinib-resistant *Kras*^*LA2*^ mice. (**A**–**F**) *Kras*^*LA2*^ mice were treated for 7 weeks by oral gavage with 25 mg/kg erlotinib 5 days a week for 7 weeks (4 mice for vehicle and 6 mice for erlotinib), or by intraperitoneal injection with 25 mg/kg KYA1797K 6 days a week for 7 weeks (7 mice for vehicle and 7 mice for KYA1797K). (**A**) H&E staining images of lung tumors (arrows) in mice treated with erlotinib or with KYA1797K. (**B**,**C**) The number of tumors in mice treated with erlotinib (**B**) or KYA1797K (**C**) were counted separately based on size. Tumors which sizes are less than 1 mm are counted as small, 1–3 mm as medium and greater than 3 mm as large. Error bars represent the SD; ^*^P < 0.05, ^**^P < 0.01, ^***^P < 0.001; *n* = 4 (vehicle), 6 (erlotinib) (**B**) or *n* = 7 (vehicle), 7 (KYA1797K) (**C**). (**D**) Immunohistochemical of the downstream effectors of the Wnt/β-catenin and the Ras-ERK pathways in the lung tumor regions from mice treated with erlotinib or KYA1797K. Nuclei were counter stained with DAPI. Scale bars, 20 μm. (**E**,**F**) WCLs of lung tumors of mice treated with erlotinib (**E**) or KYA1797K (**F**) were subjected to the immunoblot analyses using indicated antibodies.

We examined the *in vivo* antitumor effects of erlotinib and KYA1797K using *Kras*^*LA2*^ mouse model^22^. The representative H&E images of *Kras*^*LA2*^ mouse lung after erlotinib or KYA1797K treatment are as shown (Fig. 6A). Erlotinib treatment did not affect the number of tumors formed in *Kras*^*LA2*^ mice (Fig. 6B). However, KYA1797K treatment decreased the number of tumors by 51% (Fig. 6C) in agreement with the cell-based *in vitro* results. There was no observed difference in the body weight between mice treated with vehicle and those treated with either erlotinib or KYA1797K (Supplementary Figs S5A and S5B). In addition, no signs of necrosis or liver fibrosis were observed from H&E and Sirius red staining of liver tissues of mice treated with drugs (Supplementary Figs S5C and S5D). We analyzed various organs of mice treated with KYA1797K by H&E staining to ensure that KYA1797K does not induce organ toxicity and no sign of toxicity was observed in the organs analyzed. Also, there was no noticeable histological difference between the organs of KYA1797K-treated mice and those of vehicle-treated mice (Supplementary Fig. S6). Although level of p-EGFR was significantly reduced in erlotinib-treated *Kras*^*LA2*^ mice, the activities of ERK and ATF2, which is an ERK downstream transcriptional factor, were unaffected by erlotinib treatment (Fig. 6D,E). In contrast, both ERK and ATF2 activities were inhibited in lung tumors of mice treated with KYA1797K. In addition, pan-Ras and β-catenin were significantly reduced by KYA1797K treatment (Fig. 6D,F). Immunofluorescent staining and immunoblot of PCNA showed that KYA1797K effectively suppressed proliferation in tumor tissue in contrast to erlotinib (Fig. 6D–F). We observed decrease of p-EGFR by KYA1797K treatment in lung tumors of *Kras*^*LA2*^ mice. It is possibly due to the decrease in the level of EGFR by KYA1797K treatment (Fig. 6D,F), as observed in *in vitro* assay (Fig. 2D).

## Discussion

Ras proteins are a subfamily of small GTPases and play a key role in the regulation of the Raf-MEK-ERK and the PI3 kinase-Akt pathways. The three canonical members of Ras (H-Ras, N-Ras, and K-Ras) play roles for a wide range of physiological phenomena including proliferation, apoptosis, cell differentiation and tissue development^30^. While wild-type Ras plays an important role in many critical physiological responses as it maintains homeostasis of its activation level by its intrinsic GTPase activity, oncogenic Ras, locked in its GTP-bound activated form as a result of mutation, contributes cells to undergo uncontrolled proliferation leading to development of cancer.

*KRAS* mutation is one of the most common mutations found in many types of cancers. Ras proteins, including K-Ras, are directly or indirectly related with cancer and are therefore one of the most attractive targets for the treatment of cancers. However, most of the efforts to control Ras including development of the farnesyltransferase inhibitors, which aimed to control Ras activity by inhibiting membrane localization of Ras, were failed^31^. Even after decades-long failure of development of anti-cancer drugs targeting Ras, the importance of Ras as a therapeutic target has not diminished, if not increased, as evidenced by the second round re-spotlights^32,33^.

Since *KRAS* mutation accounts for more than 20% of all NSCLC and RAS is overexpressed in NSCLC patient tissues, we investigated the effect of KYA1797K, a small molecule which had an anti-cancer effect in CRC via degrading both β-catenin and Ras^14^, in *KRAS* mutated NSCLC. The development of KYA1797K as a Ras destabilizing compound was initiated based on series of our previous studies of the Wnt/β-catenin and the Ras-ERK pathway cross-talk^34–38^; especially, the study of Jeong *et al*. identified Ras degradation mechanism through negative regulation of the Wnt/β-catenin signaling which involves the GSK3β-mediated phosphorylation of Ras followed by recruitment of β-TrCP E3 ligase linker for polyubiquitination of Ras^34^. KYA1797K directly interacts with the RGS domain of Axin and activates GSK3β by enhancing the β-catenin destruction complex formation^14^. Thus, KYA1797K exerted its anti-cancer effect by enhancing GSKβ-mediated phosphorylation and subsequent degradation of both β-catenin and Ras in CRC^14^. KYA1797K displayed favorable pharmacological properties in pharmacokinetic analysis for Sprague-Dawley rats (Supplementary Fig. S7).

Although both *APC* and *β-catenin* mutations are rare in lung cancer^39–41^, the Wnt/β-catenin pathway is aberrantly activated in NSCLC due to abnormalities of various components of this pathway. Overexpression of activators of the Wnt/β-catenin pathway such as Wnt and Dishevelled^42,43^ and downregulation of antagonists of the pathway such as DKK3 and Wnt inhibitory factor^44,45^ were identified to be factors contributing to the activation of the Wnt/β-catenin pathway in NSCLC. By monitoring the status of downstream effectors of the Wnt/β-catenin and the Ras-ERK pathways in patient tissues, we confirmed the importance of the therapeutic approach targeting both pathways in NSCLC. Using various NSCLC cell lines with different *KRAS* mutational status and adapting *Kras*^*LA2*^ mouse model, we then demonstrated the effectiveness of KYA1797K, which destabilizes Ras via inhibition of the Wnt/β-catenin pathway, in erlotinib-resistant *KRAS* mutant NSCLC *in vitro* and *in vivo*. This study shows that the approach of controlling Ras activity by promoting its proteosomal degradation via inhibition of the Wnt/β-catenin pathway can be an effective targeted therapeutic strategy for the treatment of *KRAS*-mutated NSCLC.

## Materials and Methods

### Reagents

The antibodies used in this study were anti-p-ATF2 (T71) (Cell Signaling Technology); p-EGFR (Y1068) (Cell Signaling Technology); p-ERK (T202/Y204) (Cell Signaling Technology); α-tubulin (Calbiochem); β-catenin (BD Biosciences and Santa Cruz Biotechnology); caspase-3 (Santa Cruz Biotechnology); EGFR (Cell Signaling Technology); ERK1 (Santa Cruz Biotechnology); PARP-1/2 (Santa Cruz Biotechnology); PCNA (Santa Cruz Biotechnology); anti-Ki67 (Abcam); anti-Pan-Ras (Millipore); anti-rabbit AlexaFluor 488 (Life Technologies). Erlotinib (Tarceva®) was purchased from Cayman. KYA1797K was described in our previous study^14^. Cycloheximide was purchased from Sigma-Aldrich.

### Cell cultures

HEK293 cells and HEK-TOP cells were cultured in DMEM (Gibco) containing 10% fetal bovine serum (FBS), 100 U/mL of penicillin, and 100 μg/mL of streptomycin at 37 °C. NCI-H1650^46^ and PC-9^47^ cell lines were provided by Dr. Keun Chil Park (Samsung Medical Center, Seoul, South Korea). NCI-H460 and NCI-H23^48^ cell lines were obtained from Dr. Jong Soon Kang (Korea Research Institute of Bioscience and Biotechnology, Daejeon, South Korea). All NSCLC cell lines were maintained in RPMI 1640 (Gibco) containing 10% FBS, 100 U/mL penicillin, and 100 μg/mL of streptomycin at 37 °C.

### Luciferase reporter assay

For TOPflash luciferase reporter assay, HEK-TOP cells^14^ were plated at a density of 1 × 10^5^ cells in each well of 24-well plates 1 day before KYA1797K treatment and treated with the compound for 24 hours. For Elk-1 reporter assay, HEK293 cells were seeded at a density of 3 × 10^5^ cells in each well of 6-well plates 1 day before transfection. Each transfection was performed using the following vectors: pFA2-Elk-1, pFR-Luc, and pCMV-β-galactosidase (β-gal), previously described^34^, as well as the respective control vectors. One day after transfection, EGF and KYA1797K were treated for 24 hours.

Cells were harvested with ice-cold phosphate-buffered saline (PBS) and total cell lysates were extracted with 1 × Reporter Lysis Buffer (Promega). Luciferase activities were measured using FLUOSTAR (BMG labtech) after adding 40 μl of luciferin to 10 μl of total cell lysates. β-Galactosidase activities were also measured using FLUOSTAR (BMG labtech) after adding 44 μl of ONPG, 134 μl of 0.1 mol/L sodium phosphate, and 2 μl of 100× Mg solution to 10 μl of total cell lysates. In Elk-1 reporter assay, luciferase readout was normalized to β-galactosidase activity. Relative luciferase activity was calculated by setting normalized luciferase activity of reporter alone in Wnt-3a conditioned media (Wnt3a-CM) (TOPflash assay) or with EGF treatment (Elk-1 assay) equal to 100%.

### Immunoblot analyses

Cells were seeded at a density of 2.5 × 10^5^ cells in each well of 6-well plates, incubated for 24 hours and treated with erlotinib or KYA1797K for 24 hours. Cells were lysed with RIPA buffer (50 mmol/L Tris-HCl pH 7.4, 150 mmol/L NaCl, 1% NP-40, 0.25% sodium deoxycholate, 1 mmol/L EDTA, 1 mmol/L NaVO4, and 1 mmol/L NaF) and centrifuged at 14,000 × g for 30 minutes at 4 °C. Aliquots of lysates were subjected to the immunoblot analyses by using anti-p-EGFR (Y1068), -p-ERK (T202/Y204), -α-tubulin, -β-catenin, -caspase-3, -EGFR, -ERK1, -PARP-1/2, PCNA, or -Pan-Ras antibody.

### Real time reverse transcription-PCR

Total RNA was prepared using a TRIzol reagent according to the manufacturer’s instructions (Invitrogen). cDNA was synthesized from 2 μg of the total RNA using 200 U of M-MLV reverse transcriptase (Invitrogen). The resulting cDNA (1 μl) was amplified in 10 μl of 2x Rotor Gene SYBR Green PCR Master Mix (Qiagen). After an initial incubation, the cDNA was denatured at 95 °C for 5 minutes, followed by 40 cycles of PCR (95 °C for 5 seconds; 60 °C for 10 seconds). *β-Actin* was used as the endogenous control in the comparative cycle-threshold (C_T_ method). The sequences of the primers used for real-time PCR were as followed: forward (*β-Actin*) 5′-AAT CTG GCA CCA CAC CTT CTAC-3′; reverse (*β-Actin*) 5′-ATA GCA CAG CCT GGA TAG CAA C-3′; forward (*β-Catenin*) 5′-ACA AGC CAC AAG ATT ACA AGA A-3′; reverse (*β-Catenin*) 5′-GCA CCA ATA TCA AGT CCA AGA-3′; forward (*HRAS*) 5′- GGA AGC AGG TGG TCA TTG-3′; reverse (*HRAS*) 5′-AGA CTT GGT GTT GTT GAT GG-3′; forward (*KRAS*) 5′-AAA CAG GCT CAG GAC TTAG-3′; reverse (*KRAS*) 5′-GTA TAG AAG GCA TCA TCA AC-3′; forward (*NRAS*) 5′-AAG AGT TAC GGG ATT CCA TTC-3′; reverse (*NRAS*) 5′-CCA TCA TCA CTG CTG TTG A-3′.

### Colony formation assay

Cells were seeded at a density of 500–1000 cells in each well of 6-well plates and transformation assay was performed as described previously^14^. The cells were replaced with new media mixed with erlotinib or KYA1797K every 2 to 3 days for 10 to 21 days. After harvest, cells were stained with 0.5% crystal violet in 20% ethanol.

### MTT assay

Cells were seeded at a density of 1.5 × 10^4^ cells in each well of 24-well plates and treated with erlotinib or KYA1797K for 0, 24, 48, and 72 hours. MTT reagent (3-(4,5-Dimethylthiazol-2-yl)-2,5-diphenyltetrazolium bromide; AMRESCO) was diluted in RPMI 1640 at a concentration of 0.5 mg/mL and was treated with cells for 2 hours at 37 °C. Media was removed and insoluble purple formazan was solubilized with 1 mL DMSO for 30 minutes. The absorbance of formazan product was determined at 590 nm. Each experiment was repeated three times. In order to acquire IC_50_ values of KYA1797K treatment in different cell lines, the same procedure was performed except for the difference in cell density and treatment time. NCI-H1650, PC-9, NCI-H460, NCI-H23, and A549 cells were seeded at a density of 8.0 × 10^3^, 1.0 × 10^4^, 8.0 × 10^3^, 6.0 × 10^3^, and 1.5 × 10^4^ cells in each well of 96-well plates, respectively, and the all cells were treated with KYA1797K for 72 hours.

### Immunocytochemistry

Cells were fixed in 4% paraformaldehyde in PBS mixture for 30 minutes at room temperature and washed with PBS. For permeabilization, the cells were treated with 0.2% Triton-X-100 for 15 minutes at room temperature and blocked with 5% bovine serum albumin (BSA) and 1% normal goat serum in PBS for 30 minutes at room temperature followed by incubation with anti-Ki67 antibody (1:500), overnight at 4 °C. Then, the cells were incubated with an anti-rabbit Alexa Fluor 488 secondary antibody, counterstained with DAPI (Boehringer Mannheim), and mounted in Gel/Mount media (Biomeda Corporation). Images were taken using confocal microscopy (LSM510META and LSM700, Carl Zeiss). Each experiment was repeated three times.

### Caspase 3/7 activity assay

Cells were seeded at a density of 2 × 10^4^ cells in each well of 96-well plates and treated with erlotinib or KYA1797K for 36 hours. The treated cells were subjected to Caspase 3/7 activities measurement with Caspase-Glo 3/7 assay kit (Promega). After the plates containing cells were allowed to equilibrate to room temperature, Caspase-Glo reagent (100 μl) was added to each well in a 1:1 ratio with media and the content of well was gently mixed at a shaker for 30 seconds. The plate was then incubated at room temperature for 1 hour. The luminescence of each sample was measured using FLUOSTAR. Each experiment was repeated three times.

### Cell cycle and apoptosis assays

NCI-H1650 and NCI-H460 cells were seeded at a density of 5 × 10^5^ cells in 60 mm dish and treated with 1 μmol/L erlotinib or 25 μmol/L KYA1797K for 48 hours. For analysis of cell cycle and apoptosis, cells were harvested and fixed with 75% ethanol for 1 hour at 4 °C. The fixed cells were stained with 50 μg/ml PI (Sigma-Aldrich) in 3.8 mmol/L sodium citrate buffer for 2 hours and analyzed by BD FACSCalibur (BD Biosciences) to assess the number of apoptotic cells (sub-G1) and the cell cycle distribution according to DNA content. For double staining with PI and annexin V-FITC (Sigma-Aldrich), cells were harvested and suspended in binding buffer (10 mmol/L HEPES, 140 mmol/L NaCl, and 2.5 mmol/L CaCl_2_) at a density of 1 × 10^6^ cells/ml. One hundred micro liters of the cell suspension were transferred into a 5 ml culture tube and incubated with 5 µl annexin V-FITC and 10 µl PI for 15 minutes in the dark. A volume of 400 µl of binding buffer was added to each tube, and the cells were analyzed by BD FACSCalibur (BD Biosciences). Due to the fluorescence of KYA1797K, quadrant lines for KYA1797K-treated samples were adjusted by using unstained KYA179K-treated cells as a control.

### Animal experiment

All animal experiments were performed in accordance with the Korean Food and Drug Administration guidelines. Protocols were reviewed and approved by the Institutional Animal Care and Use Committee (IACUC) of Yonsei University. B6.129S-*Kras*^*tm3Tyj*^ (*Kra*^*LA2*^) mice^38^ were obtained from National Cancer Institute mouse repository and were housed in filter-topped shoebox cages in a pathogen-free facility at Yonsei University (Seoul, South Korea). Erlotinib, in a suspension of 1% methylcellulose and 0.2% Tween 80, was injected by oral gavages at dosage of 25 mg/kg, 5 days a week (4 mice for vehicle and 6 mice for erlotinib) for 7 weeks. KYA1797K, dissolved in PBS mixed with 0.5% DMSO and 5% Tween 80, was intraperitoneally treated to mice at dosage of 25 mg/kg, 6 days a week for 7 weeks (7 mice for vehicle and 7 mice for KYA1797K). Mice were weighed once a week. Immediately after sacrifice, the abdomens of the mice were cut open longitudinally and cleaned by flushing with PBS. Lung tissues were dissected and fixed in 4% neutral paraformaldehyde (PFA).

### H&E and Sirius red staining

The dissected tissues were fixed in 4% PFA and embedded in paraffin. The paraffin sections were cut at a thickness of 4 μm and stained with hematoxylin and eosin (H&E) or with picro-sirius red. Pictures of stained tissues were taken using a Nikon bright-field optical microscope (Nikon TE-2000U).

### Immunohistochemistry

For fluorescent immunohistochemical analysis, 4 μm paraffin sections were deparaffinized and rehydrated. Then the sections were treated with 0.1% triton X-100 in PBS for 15 minutes, followed by blocking with 5% BSA and 1% NGS in PBS for 30 minutes. Primary and secondary antibodies were diluted with 1% BSA and 1% NGS in PBS. Sections were incubated with primary antibodies, overnight at 4 °C, followed by incubation with anti-mouse Alexa Flour 488 (1:500) or anti-rabbit Alex Flour 555 secondary antibody (1:500) for 1 hour at room temperature. Nuclei were counter stained by DAPI, and the sections were mounted with Gel/Mount media (Biomeda Corporation). All incubations were carried out in dark and humid chambers. The fluorescence signal was visualized using a confocal microscopy (LSM510) at excitation wavelengths of 488 nm (Alexa Fluor 488), 543 nm (Alexa Fluor 555), and 405 nm (DAPI).

For 3,3′ Diaminobenzidine (DAB) immunohistochemical analysis, 4 μm paraffin sections were deparaffinized and rehydrated. The slides were autoclaved in 10 mmol/L sodium citrate buffer (pH 6.0) for antigen retrieval. The sections were blocked in 5% BSA in PBS at room temperature for 1 hour. The sections were incubated with primary antibody overnight at 4 °C followed by biotin-conjugated secondary antibodies for 1 hour at room temperature. The sections were incubated with an Avidin-Biotin complex (Vector Laboratories) for 30 minutes to 1 hour followed by DAB staining (Vector Laboratories). The DAB stained preparations were visualized with a bright-field optical microscope (Nikon TE-2000U). At least 3 fields per section were analyzed to establish statistical significance.

### Statistical analyses

Data are expressed as means and the standard deviation (SD) or the standard error mean (SEM). Student *t* test was used to determine differences between groups. All statistical tests were two-sided and *P* values less than 0.05 were considered statistically significant.

## Supplementary information

Supplementary information
