# Supplementary material for: A Ras destabilizer KYA1797K overcomes the resistance of EGFR tyrosine kinase inhibitor in KRAS-mutated non-small cell lung cancer
Source: Sci Rep. 2019 Jan 24;9:648. doi: 10.1038/s41598-018-37059-8 (PMC6345925; doi:10.1038/s41598-018-37059-8)
Supplement: Supplementary file 1 — Supplementary information [file 41598_2018_37059_MOESM1_ESM.docx]

**Supplementary information**

**A small molecular RAS destabilizer overcomes the resistance of EGFR tyrosine kinase inhibitor in *KRAS*-mutated non-small cell lung cancer**

Jieun Park^1,2^, Yong-Hee Cho^1,2^, Wook-Jin Shin^1,2^, Sang-Kyu Lee^1,2^, JaeHeon Lee^1,2^, Tae Hyung Kim^1,2^, Pu-Hyeon Cha^1,2^, Jee Sun Yang^1,2^, Jaebeom Cho^4^, Do Sik Min^1,3^, Gyoonhee Han^1,2^, Ho-Young Lee^4^, and Kang-Yell Choi^1,2^

^1^Translational Research Center for Protein Function Control, Yonsei University, Seoul, Korea. ^2^Department of Biotechnology, College of Life Science and Biotechnology, Yonsei University, Seoul, Korea. ^3^Department of Molecular Biology, College of Natural Science, Pusan National University, Pusan, Korea. ^4^Creative Research Initiative Center for Concurrent Control of Emphysema and Lung Cancer, College of Pharmacy, Seoul National University, Seoul, Korea. Correspondence and requests for materials should be addressed to K-Y.C. (email: [kychoi@yonsei.ac.kr](mailto:kychoi@yonsei.ac.kr))

**Supplementary Table S1**

**
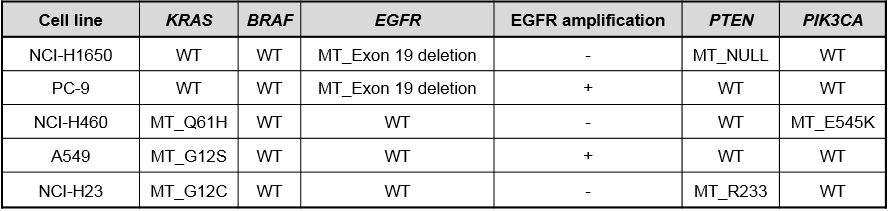
**

**Supplementary Table S1.** Abberrancies fo various NSCLC cell lines

**Supplementary Figure S1**

**
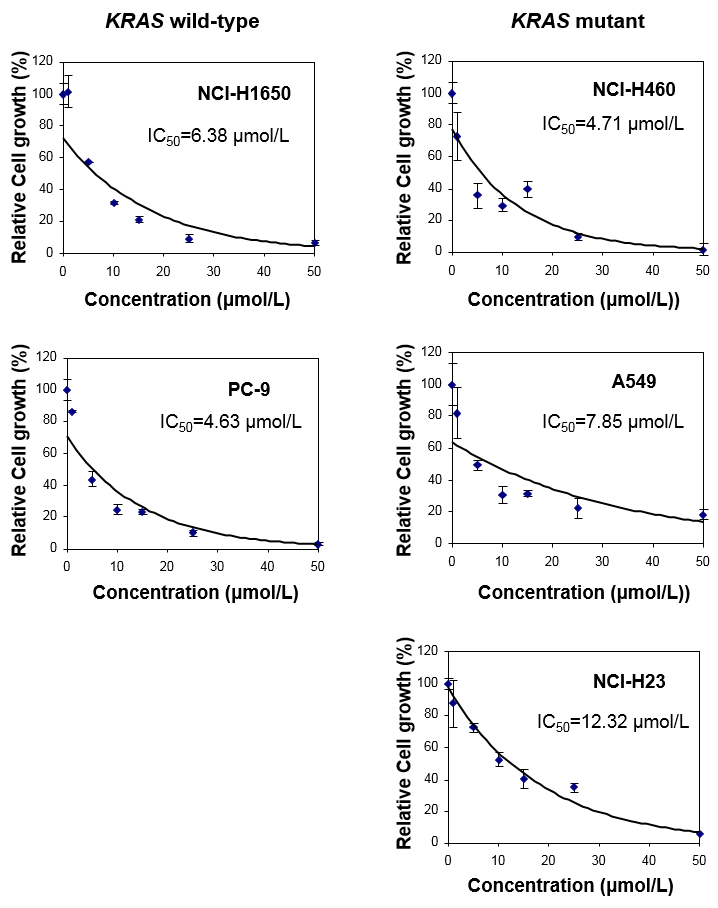
**

**Supplementary Figure S1.** Effects of KYA1797K on the growth of NSCLC cells. IC_50_ values of KYA1797K in *KRAS* wild-type and mutant NSCLC lines. NSCLC cells were treated with varying concentrations of KYA1797K for 72 hours. MTT assays were performed and error bars represent the SD; *n* = 3.

**Supplementary Figure S2**


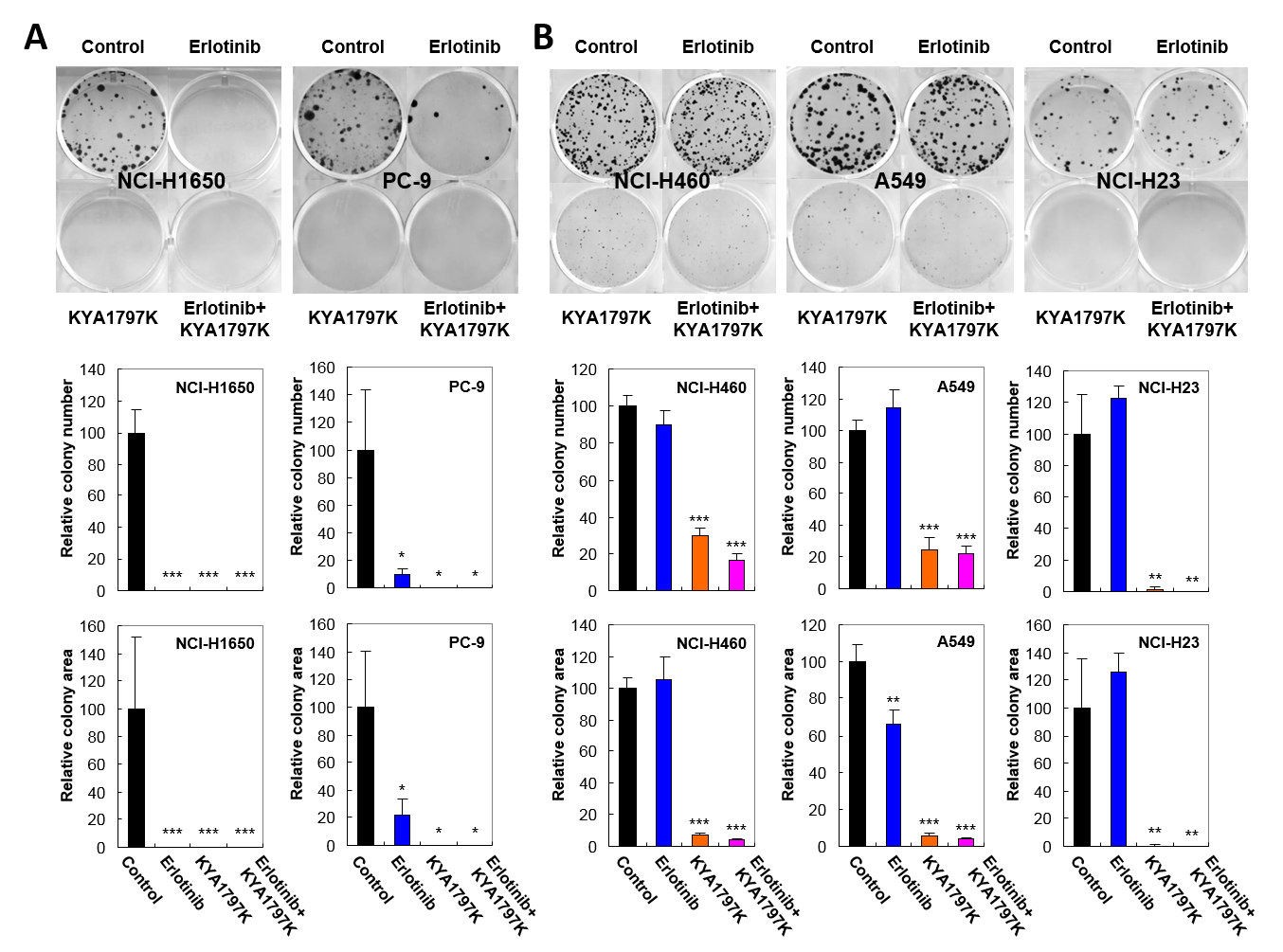


**Supplementary Figure S2.** Effects of combined treatment of erlotinib and KYA1797K on cellular transformation of *KRAS* wild-type and *KRAS* mutant NSCLC cell lines. A and B *KRAS* wild-type (**A**) and mutant NSCLC cell lines (**B**) were treated every 2 or 3 days with 1 μmol/L erlotinib, 25 μmol/L KYA1797K, or 1 μmol/L erlotinib in combination with 25 μmol/L KYA1797K for 10-21 days. Cells were stained with 0.5% crystal violet, and then foci were photographed. Quantitative data for the relative percentages of colony numbers and areas are determined with ImageJ software and normalized by DMSO-treated control. Data represented mean ± SD; ^*^*P* < .05, ^**^*P* < .01, ^***^*P* < .001; *n* = 3.

**Supplementary Figure S3**


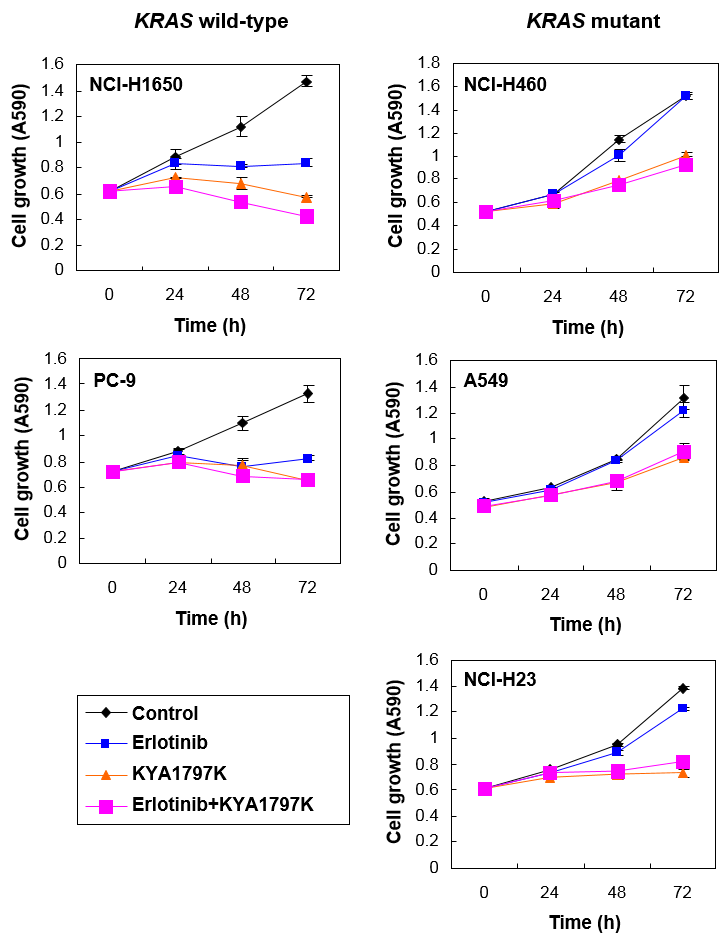


**Supplementary Figure S3.** Effects of combination treatment of erlotinib and KYA1797K on cell growth of *KRAS* wild-type and mutant NSCLC cell lines. NSCLC cells were treated with 1 μmol/L erlotinib, 25 μmol/L KYA1797K or 1 μmol/L erlotinib in combination with 25 μmol/L KYA1797K to NSCLC cell lines. MTT assays were done at 0, 24, 48, and 72 hours after erlotinib or KYA1797K treatments. Error bars represent the SD; *n* = 3.

**Supplementary Figure S4**

**
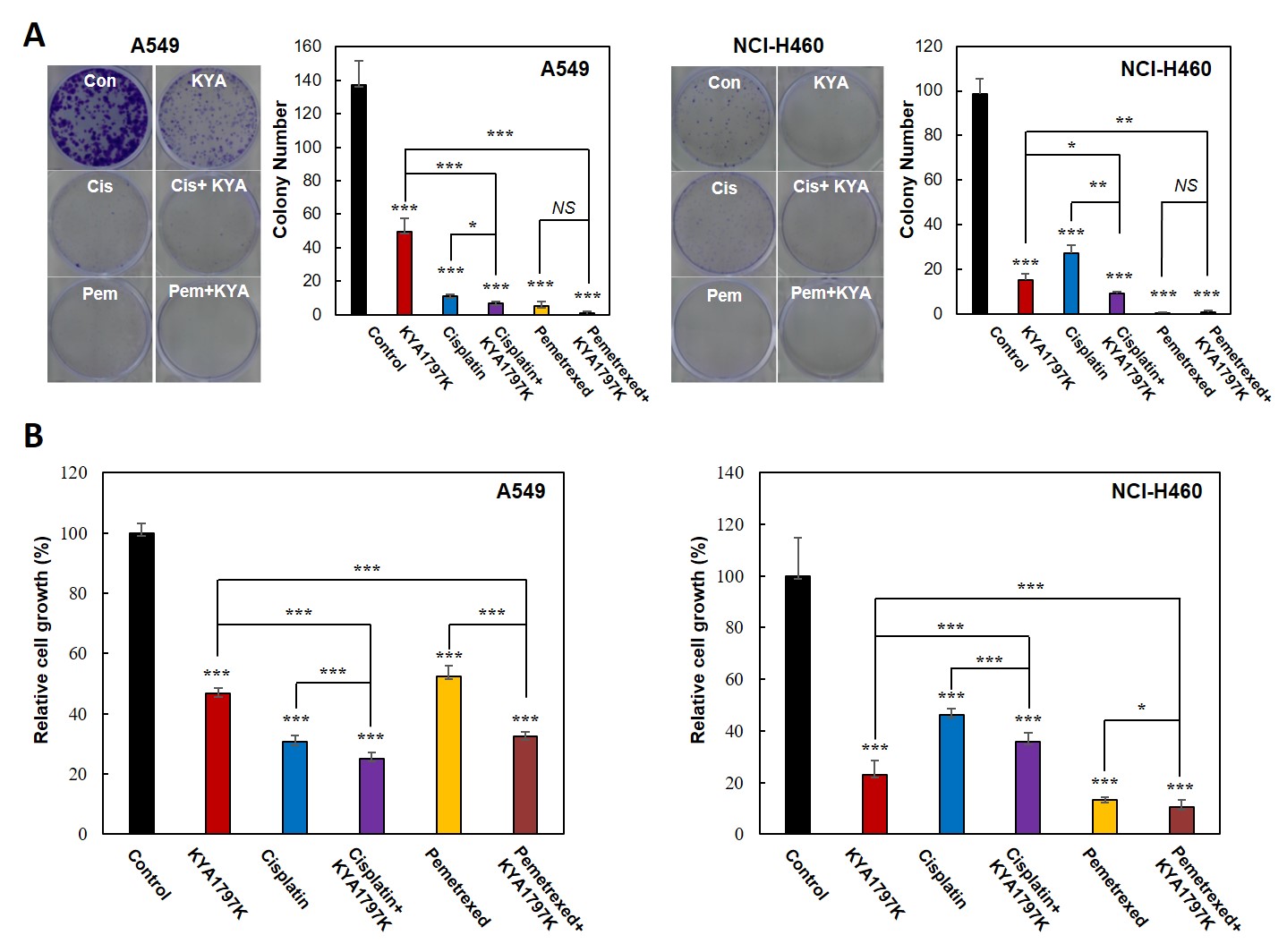
**

**Supplementary Figure S4.** Comparison of the efficacy of KYA1797K vs. cisplatin or pemetrexed on colony formation capacity and cell growth of *KRAS* mutant A549 and H460 cell lines. **A**, NSCLC cells were treated every 2 or 3 days with 5 μmol/L cisplatin, 5 μmol/L pemetrexed, 25 μmol/L KYA1797K or 5 μmol/L cisplatin or pemetrexed in combination with 25 μmol/L KYA1797K for 10-21 days. Cells were stained, imaged and quantified as described previously. Data represented mean ± SD; ^*^*P* < .05, ^**^*P* < .01, ^***^*P* < .001; *n* = 3. **B,** NSCLC cells were treated with 5 μmol/L cisplatin, 5 μmol/L pemetrexed, 25 μmol/L KYA1797K or 5 μmol/L cisplatin or pemetrexed in combination with 25 μmol/L KYA1797K and MTT assays were done at 72 hours after drug treatment. Data represented mean ± SD; ^*^*P* < .05, ^**^*P* < .01, ^***^*P* < .001; *n* = 6.

**Supplementary Figure S5**


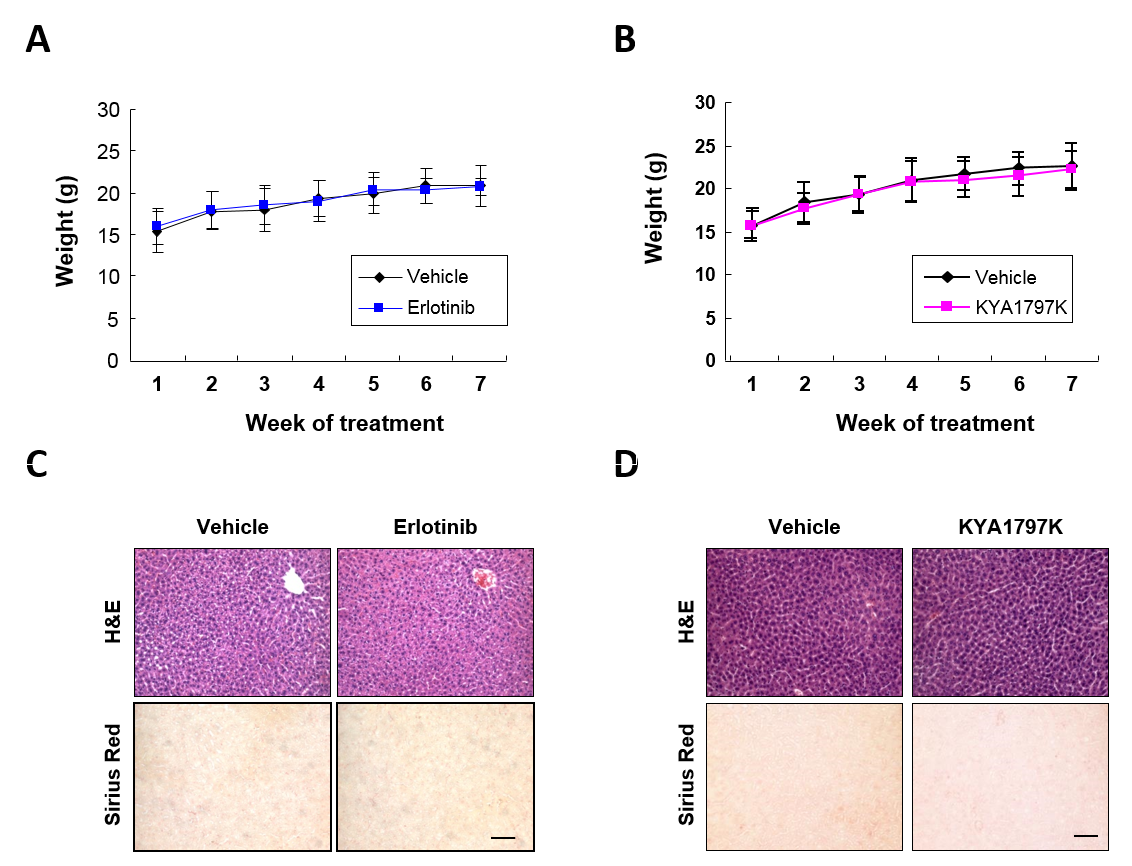


**Supplementary Figure S5.** Effects of KYA1797K on weight and liver of K*ras* mutated mice. No sign of liver toxicity was observed in liver tissues of *Kras^LA2^* mice treated with 25 mg/kg of erlotinib or KYA1797K. (**A**,**B**) the body weight of mice treated with erlotinib (**A**) or KYA1797K (**B**) were measured once a week. The error bars represent the SD; *n* = 4/6 per group (**A**) or *n* = 7/7 per group (**B**). (**C**,**D**) H&E (Top) and Sirius red (bottom) staining images of liver tissues of *Kras^LA2^* mice treated with erlotinib (**C**) or KYA1797K (**D**). Scale bars, 40 μm.

**Supplementary Figure S6**


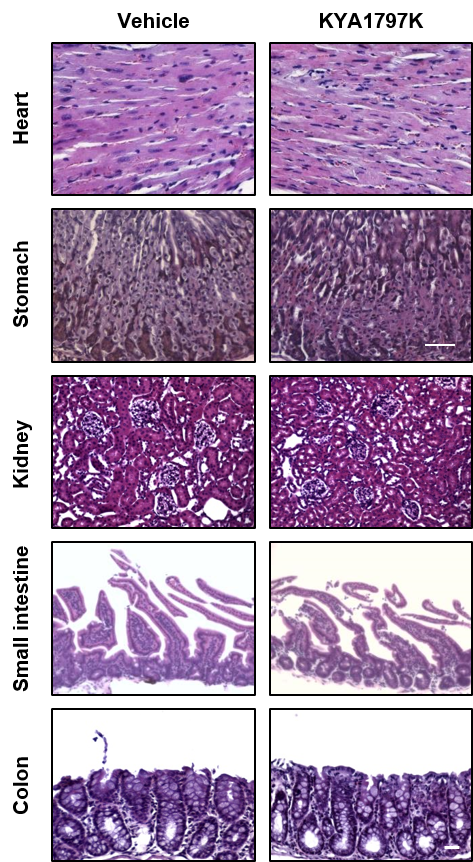


**Supplementary Figure S6.** Effects of KYA1797K on histomorphological change (organ toxicity) of *Kras* mutated mice. *Kras^LA2^* mice were treated with vehicle or KYA1797K for 7 weeks. Heart, stomach, kidney, small intestine, and colon were isolated after sacrifice, and histological sections were stained with H&E. Scale bar, 20 μm for heart, and stomach images; Scale bar, 20 μm for kidney, small intestine, and colon images.

**Supplementary Figure S7**


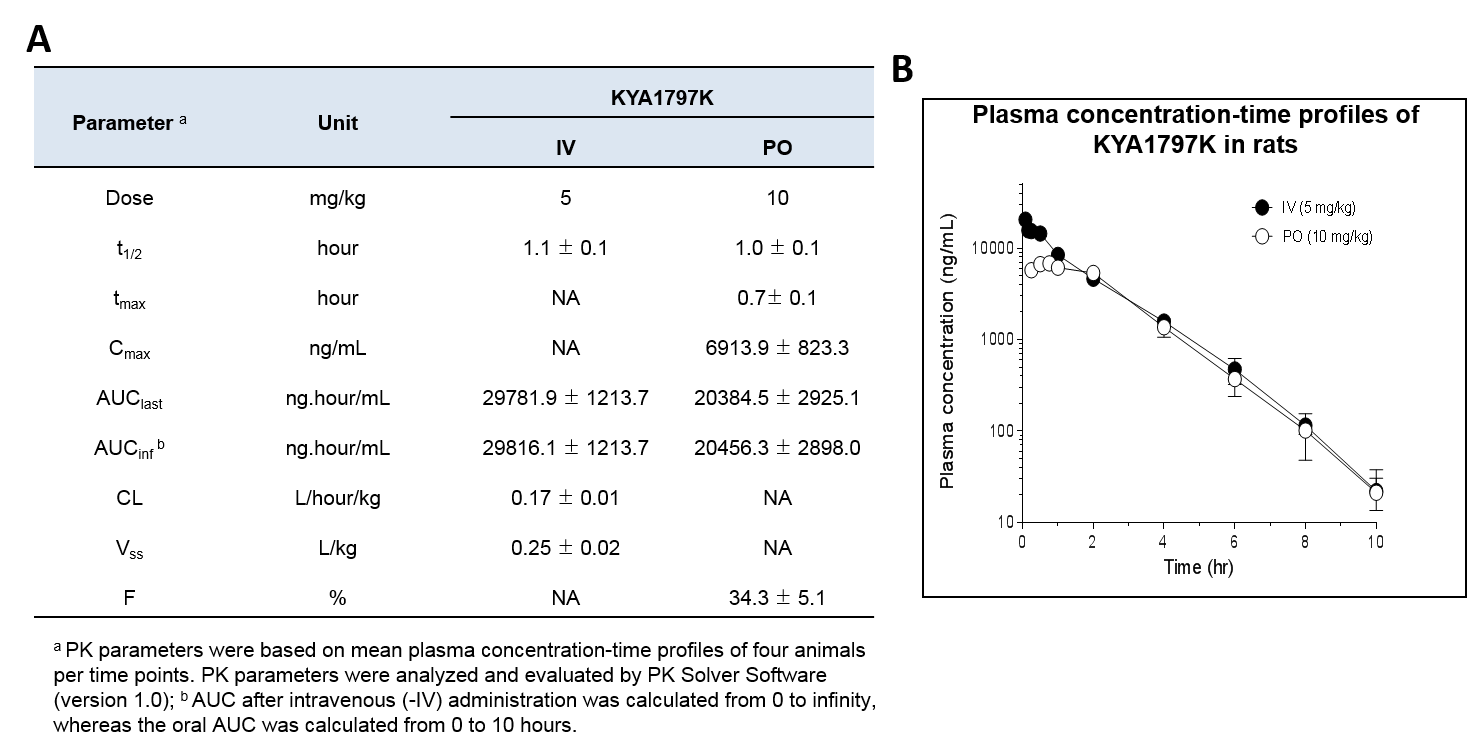


**Supplementary Figure S7.** Pharmacokinetic analysis of KYA1797K. (**A**,**B**) KYA1797K was intravenously (IV) and orally (PO) administered at 5 mg/kg and 10 mg/kg dose to male Sprague-Dawley rats. Pharmacokinetic parameters (**A**) and Plasma concentration-time profiles (**B**) of KYA1797K in rats are presented. Data represent mean ± SD.

**Note for Supplementary Figure S7.** To investigate pharmacokinetic effects of KYA1797K *in vivo*, KYA1797K was intravenously and orally administered at 5 mg/kg and 10 mg/kg dosages, respectively, to male Sprague-Dawley rats. The systemic exposure, clearance, and steady state volume of distribution of KYA1797K were 29781.9 ng∙hour/ml, 0.17 l/hour/kg, and 0.25 l/kg, respectively (Figure S7). After oral administration, KYA1797K also exhibited high plasma exposure at 20456.3 ng∙hour/ml. Moreover, the oral bioavailability of KYA1797K was 34.3% at 10 mg/kg oral dose. KYA1797K showed significant plasma exposure and tolerable oral bioavailability (Figure S7). The terminal half-lives of KYA1797K were approximately 1.1 hour after intravenous administration and 1.0 hour after oral administration (Figure S7).
